# Household cooking fuel use and its health effects among rural women in southern India—A cross-sectional study

**DOI:** 10.1371/journal.pone.0231757

**Published:** 2020-04-27

**Authors:** Beulah Sarah James, Ranjitha S. Shetty, Asha Kamath, Avinash Shetty

**Affiliations:** 1 Epidemic Diseases Hospital, Mysuru, Karnataka, India; 2 Department of Community Medicine, Manipal Academy of Higher Education, Kasturba Medical College Manipal, Manipal, Karnataka, India; 3 Department of Data Science, Manipal Academy of Higher Education, Prasanna School of Public Health, Manipal, Karnataka, India; Valahia University of Targoviste, ROMANIA

## Abstract

The use of biomass fuel is associated with the deterioration of human health and women are more likely to develop health conditions due to their exposure to indoor air pollution during cooking. This study was conducted to assess the pattern of fuel used for cooking in households as well as to determine the association between the types of fuel used with respect to socio-demographic characteristics and health status of women. A community based cross-sectional survey was conducted between August 2016 and September 2018 in four rural areas and one semi-urban area of Udupi district, Karnataka, India. The study comprised 587 families including 632 women. A pre-tested semi-structured questionnaire was used to collect data on the type of fuel as well as self-reported health conditions. Overall, 72.5% of the families used biomass, where 67.2% families were currently using both biomass and liquefied petroleum gas while only biomass was used in 5.3% of the families for cooking. Among women, being ever exposed to biomass fuel was significantly associated with their age, literacy level, occupation and socio-economic status (p < 0.001). Those who were exposed to biomass fuel showed a significant association with self-reported ophthalmic (AOR = 3.85; 95% CI: 1.79–8.29), respiratory (OR = 5.04; 95% CI: 2.52–10.07), cardiovascular (OR = 6.07; 95% CI: 1.88–19.67), dermatological symptoms /conditions (AOR = 3.67; 95% CI: 1.07–12.55) and history of adverse obstetric outcomes (AOR = 2.45; 95% CI: 1.08–5.57). A positive trend was observed between cumulative exposure to biomass in hour-years and various self-reported health symptoms/conditions (p < 0.001). It was observed that more than two-thirds of women using biomass fuel for cooking were positively associated with self-reported health symptoms. Further longitudinal studies are essential to determine the level of harmful air pollutants in household environment and its association with various health conditions among women in this region.

## Introduction

Indoor air pollution (IAP) is one of the world's major environmental problems. It is mainly caused by the use of solid fuels for cooking which includes biomass (e.g. wood, crop residues, animal dung, and charcoal) and coal. IAP refers to the contamination of air inside the house by solid fuel combustion for any purpose leading to indoor air pollution as well as contributing to ambient air pollution. The Institute for Health Metrics and Evaluation (IHME) indicates that about 2.6 million people died prematurely in 2016 from diseases attributable to IAP globally [1].

According to the Global Burden of Disease Report, IAP is the leading cause of disability-adjusted life years (DALYs) in Southeast Asia and the third leading cause of DALYs worldwide [2]. It is considered a silent killer that has resulted in 4.3 million deaths worldwide accounting for 7.7% of the global mortality. The South-East Asian region contributes to the maximum mortality due to indoor air pollution followed by the Western Pacific region [3]. The key health conditions that attributes to the global mortality due to IAP are pneumonia, stroke, ischemic heart disease (IHD), chronic obstructive pulmonary disease (COPD), lung cancer and poor obstetric outcomes [4]. Existing literature suggests that the statistics on access to clean fuels for cooking in a given region can be used as a surrogate indicator of disease risk [5]^.^

World Health Organization (WHO) estimates that about 3 billion people use open fire or traditional stoves that are fuelled by kerosene and solid fuels, globally [6]. People from low socio-economic background are forced to use solid fuels as these are available easily in rural areas at a lower cost [7, 8]. According to the Tracking SDG7: The Energy Progress Report 2018, if the current pattern continues, 2.3 billion people would be still using biomass in 2030 globally [9].

In India, approximately 64% of the households use solid fuels. There is a contrast between the rural and urban areas with 81% of the rural households using solid fuels as compared to only 26% of their urban counterparts [10]. However, according to the National Family Health Survey-IV (NFHS-IV) conducted between 2015 and 2016, the total number of households using clean fuel has doubled to 43.8% compared to the mere 25.5% in the previous (NFHS-III) survey conducted between 2005 and 2006 [11]. This trend confirms the energy ladder pattern where biomass is used in lower-income families and a shift towards cleaner fuels is observed with increasing income of the families [11].

Women and children under five years who spend most of their time at home are majorly affected by IAP [12]. Women in India are traditionally responsible for performing household chores and cooking, which results in spending several hours close to the cooking fire being exposed to the hazardous effects of smoke. Biomass fuels that are commonly used in Indian households tend to produce more organic compounds such as benzene, formaldehyde, 1, 3-butadiene, polyaromatic hydrocarbons etc. and their concentrations remain higher when cooking is done indoor without proper smoke outlets [5]. This would result in eye irritation and watering, respiratory problems, poor obstetric outcomes and burns [13]. Balakrishnan K. had summarized the mortality estimates due to IAP in recent research studies attributing 20% of ischemic heart diseases (IHD), 23% of stroke, 45% of chronic obstructive pulmonary disease (COPD), 21% of lung cancer and 22% of acute respiratory tract infections (ARI) to IAP [14]. Similarly, many studies conducted across India have reported the positive association between biomass fuel use and different health conditions among women ranging from throat irritation, allergic rhinitis, COPD, hypertension, severe burns and poor obstetric outcomes [15, 16, 17, 18, 19, 20, 21, 22, 23, 24 and 25].

According to the 2011 sub-district census data of Udupi, 70.3% of rural households still utilize biomass fuel for cooking despite Udupi being one of the developed districts of Karnataka state with respect to literacy levels and socio-economic status of the population [26]. Of these, many are following a multiple fuels strategy, i.e. using both biomass fuel and liquefied petroleum gas (LPG). The reason for such a practice is that parboiled rice is being used as the staple diet in the coastal regions, which takes longer time (approximately 1–2 hours) to be cooked properly. Because LPG cylinders do not last for an entire month if they are used for cooking parboiled rice daily, people prefer to use biomass fuel as an alternative source, which is less expensive and easily available.

Despite the widespread utilization of biomass fuel, there is a paucity of literature on exposure to biomass fuel and its health effects among women in this part of the state. It is worth to mention here that the Sustainable Development Goals (SDGs) 2015 emphasize on the necessity to ensure access to affordable, sustainable and clean energy for all by the year 2030 [27].

The present study was carried out to determine the pattern of fuel used for cooking in rural and semi urban households and to evaluate the association of type of fuel used with socio-demographic characteristics and health status of the women in an area with relatively better socio-economic indicators.

## Materials and methods

**Fig 1:**
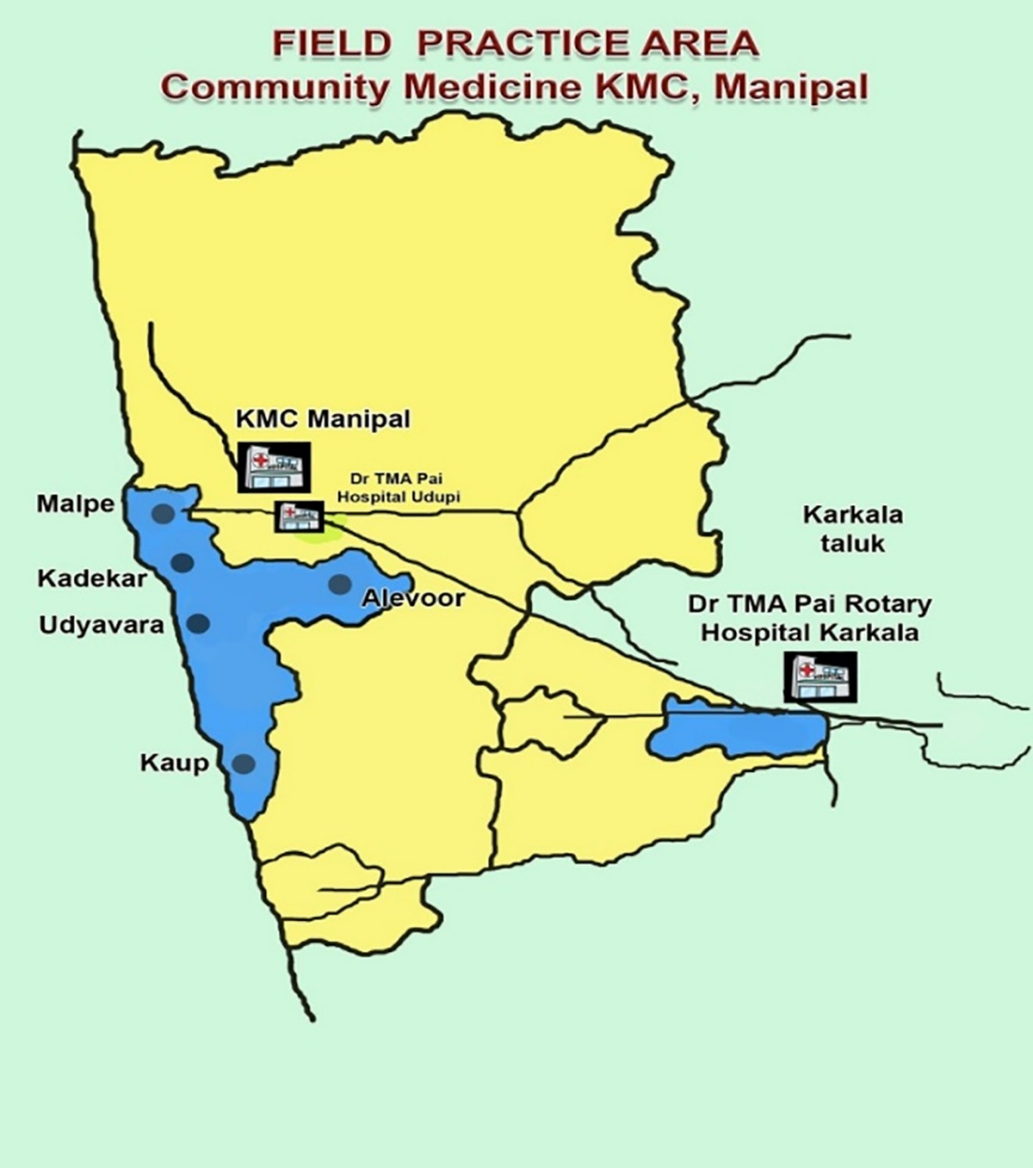
Field practice area of the department of community medicine, KMC Manipal.

A community based cross-sectional survey was carried out between August 2016 and September 2018 in the field practice area of the Department of Community Medicine, Kasturba Medical College (KMC), Manipal. The field practice area is located along the coastal belt of Udupi district, being spread across 13 villages comprising approximately 42,000 residents with 16,374 women that are aged ≥ 18 years during the study period. The department provides health care services to this population through a network of five outreach centres, which are linked to the secondary and tertiary health care facilities as illustrated in Fig 1.

Each centre has two Auxiliary Nurse Midwives (ANM), with primary care physicians providing out-patient services. In addition, health education sessions are being carried out on a regular basis on preventive aspects of health and disease for targeted groups of the communities. The detailed information of the population in the field practice area with regard to their socio-demographic profiles and health status is updated and documented periodically in the Health and Demographic Surveillance System (HDSS), which is a dynamic database of the department.

The study proposal was approved by the Institutional Ethics Committee, Kasturba Hospital, KMC Manipal, prior to initiation of the study (Registration no: ECR/146/Inst/KA/2013; Project Approval no.: IEC 592/2016). Women aged 18 years and above residing in the study area for more than a year were eligible for the study. Sick or bedridden women who were not able to respond to the questionnaire were excluded from the survey.

Considering the prevalence of firewood and kerosene use in the field practice area to be 45% (unpublished field data from the study area), at 95% confidence intervals with relative precision of 10% and a non-response rate of 20%, 587 families were needed to be recruited into the study. Households were selected based on probability proportional to size approach from the field practice area. The field practice area is representative of the population from the district as they share similar diet, socio-economic status, occupation and cooking practices.

During the house visits, the principal investigator moved in one particular direction from a randomly selected point until the proportionate sample size of each area was attained. All the eligible and consenting women from the selected houses were recruited into the study. Women from the houses, which were locked during two consecutive visits made by the investigator and those who refused to participate in the study, were considered as non-respondents.

Written informed consent was obtained from all the participants before recruiting them into the study. A pre-tested semi-structured questionnaire was developed using standard questionnaires used for assessing similar research questions in India and other developing countries [28, 29 and 30]. The questionnaire had two sections (Part 1 –Family details and Part 2—Individual details), which was administered to the eligible women in each of the selected houses to gather data on their socio-demographic details, cooking practices such as place of cooking, commonly used stoves, type of fuel used, duration of cooking etc. The socio–economic status was assessed using modified BG Prasad scale [31]. The self-reported health effects associated with specific type of household fuel use, the duration of the symptoms and whether any medical treatment was sought were enquired among women. All the explanatory variables were constructed based on existing national and international literature [28, 29 and 30]. Exposure to biomass fuel was the main outcome variable in the study and was defined as the utilization of wood, crop residues, animal dung and charcoal for household cooking purposes currently or in the past. The questionnaire was performed in the local language (Kannada) by the investigator.

Data was analysed using SPSS version 15.0 and was summarized as frequencies and percentages. Association between socio-demographic characteristics and cumulative exposure to fuel used for cooking and health effects was expressed as odds ratio (OR) with 95% confidence intervals (CI). All exogenous variables found to be predictors at univariate analysis at a p≤0.2 were further included in the final multivariable regression model. Binary logistic regression was used for each of the morbidities adjusting for multiple testing.

## Results

The survey was conducted among 587 families, which included 632 women. Of the total study population, almost half (49.5%) belonged to the age group of 18–45 years. Though most of them (63.2%) had ≥10 years of schooling, only about 17.4% of them were employed and majority (79.1%) were homemakers. Of the surveyed families, most of them belonged to Hindu religion (91.8%) and more than two-third of the families (74.8%) belonged to middle socio-economic background (Table 1).

**Table 1:** Baseline characteristics of the surveyed population (n = 632).

Characteristics		Frequency (%)
**Age group (in years)**	18–45	313 (49.5%)
	46–60	211 (33.4%)
	>60	108 (17.1%)
**Literacy status**	Illiterate	42 (6.6%)
	Primary school	60 (9.5%)
	Middle school	131 (20.7%)
	High school	203 (32.2%)
	Pre University Course	76 (12.0%)
	Graduate	105 (16.6%)
	Postgraduate	15 (2.4%)
**Occupational status**	Professional	11 (1.7%)
	White collar	22 (3.5%)
	Skilled	6 (0.9%)
	Semiskilled	36 (5.7%)
	Unskilled	10 (1.6%)
	Home maker	500 (79.1%)
	Student	22 (3.5%)
	Retired	25 (4.0%)
Religion* **(n = 587)**	Hindu	539 (91.8%)
	Christian	19 (3.3%)
	Muslim	29 (4.9%)
Socio- economic status# (n = 587)*	Upper	114 (19.4%)
	Middle	439 (74.8%)
	Lower	34 (5.8%)

# assessed using modified B.G Prasad’s scale for socio-economic classification, which is an income-based scale used in India and hence, requires constant update to take into account of inflation and depreciation of Indian rupees.

*total number of surveyed families

### Current cooking practices in the families and participants

Of the 587 families, more than half (53.1%) had a separate kitchen within their houses, 34.9% had their kitchen as a separate building outside the house, 10.6% cooked outdoors, while the rest of them used either living room or sleeping room itself for cooking.

On assessing the pattern of cooking among the study participants, majority of them (94.3%) were involved in cooking, while 24 (3.8%) women were involved in cooking in the past. However, about 12 (1.9%) women claimed to have been exposed to cooking fuel without being involved in cooking. Of those 620 women involved in cooking, 143 (23.1%) women were involved in cooking for ≤15 years with 51 (8.2%) of them being involved for less than 5 years.

### Pattern of household fuel used for cooking among the surveyed families and participants

Table 2 shows that majority (72.5%) of the families were using biomass fuel for cooking with 92.7% of them using biomass fuel and LPG simultaneously. However, 5.3% used only biomass as fuel for cooking. Further, it was noted that almost two third of the families (63.8%) with biomass stoves did not have smoke outlets.

**Table 2:** Type of fuel used for cooking among the families in the surveyed area (n = 587).

Type of fuel	Frequency (%)
Only Biomass	31 (5.3)
Biomass and LPG	395 (67.2)
Only LPG	161 (27.5)

As shown in Table 3, majority of the women (91.3%) were ever exposed to biomass fuel, of which almost two-thirds were exposed for more than 22 years (61.0%). Majority (72.7%) of them quoted affordability and availability as the main reasons for preferring biomass fuel, especially for cooking parboiled rice. The other reasons mentioned were that it is easily accessible to them (21.8%), food cooked on biomass fuel tasting better (6.2%) and it has been a routine practice to use biomass as cooking fuel in their families since many years (4.7%). Among LPG stove users in our study, more than half of the participants (55.9%) felt that LPG takes lesser time to cook while 20.5% women were of the opinion that it was a better quality fuel and 16.2% felt that it produces less smoke. Other reasons such as moving to a new house, decreased need for fuel consumption as the number of family members had reduced compared to the past, health issues among the women and recommendations by other people to use LPG were mentioned by a small proportion of women (7.4%).

**Table 3:** Characteristics of ever used cooking fuel among the study participants.

Characteristics		Frequency (%)
Type of fuel (n = 620)^*^	Biomass	566 (91.3)
	LPG	582 (93.4)
**Duration of biomass exposure in years (n = 566)**	≤10	92 (16.2)
	11–22	129 (22.8)
	>22	345 (61.0)
**Utilization of biomass per day in minutes (n = 566)**	≤50	11 (1.9)
	51–90	121 (21.4)
	>90	434 (76.7)
**Duration of LPG stove use in years (n = 582)**	≤ 10	252 (43.3)
	11–22	231 (39.7)
	>22	99 (17.0)
**Utilization of LPG stove use per day in minutes (n = 582)**	≤50	261 (44.8)
	51–90	255 (43.8)
	>90	66 (11.4)

^*^ 620 women were involved in cooking at the time of survey or in the past

### Association of socio-demographic characteristics and exposure status of women to biomass

Of the women who were aged above 45 years, 96.9% were ever exposed to biomass as compared to 82.1% of the women in the lower age group (≤ 45 years) and this association was found to be statistically significant (p<0.001). An inverse relationship was noted wherein the women who were illiterates or had <5 years of schooling, who were homemakers or engaged in unskilled jobs and those from a low socio-economic status were more likely to have been ever exposed to biomass fuel in their lifetime (p<0.001). As self-reported smoking was not observed among women, active smoking was not used as a covariate in the analysis.

### Association of being ever exposed to biomass fuel and self-reported health conditions

In this study, the self-reported health conditions among women were grouped as follows—ophthalmic conditions included diminished vision, cataract, eye irritation and watering of eyes, while respiratory conditions included throat irritation, ear pain, asthma, nasal stuffiness/ running nose, cough with/ without phlegm. Likewise, cardiovascular conditions comprised hypertension, myocardial infarction, and stroke; dermatological conditions included skin irritation, blisters due to skin burns while miscarriages, stillbirth, low birth weight and preterm deliveries were grouped as adverse obstetric outcomes.

Being ever exposed to biomass fuel was significantly associated with self-reported ophthalmic (OR = 3.90; 95% CI: 2.31–6.61), respiratory (OR = 5.04; 95% CI: 2.52–10.07), cardiovascular symptoms /conditions (OR = 6.07; 95% CI: 1.88–19.67), dermatological (OR = 3.82; 95% CI: 1.36–10.73) and history of adverse obstetric outcomes (OR = 2.85; 95% CI: 1.36–5.96) among the study participants. However, on multivariate analysis, women with ophthalmic conditions, dermatological symptoms and adverse obstetric outcomes only showed a significant association with ever exposure to biomass fuel as depicted in Table 4.

**Table 4:** Association of being ever exposed to biomass fuel and health status among the study participants (n = 632).

Self-reported symptoms		Ever exposed		p value	Crude OR (95% CI)	AOR (95% CI) *
		YES	NO			
**Ophthalmic conditions**^#^	Present	406 (71.7)	26 (39.4)	<0.001	3.90 (2.31–6.61)	3.85 (1.79–8.29)
	Absent	160 (28.3)	40 (60.6)		1	1
**Respiratory symptoms** ^$^	Present	268 (47.3)	10 (15.2)	<0.001	5.04 (2.52–10.07)	
	Absent	298(52.7)	56 (84.8)		1	
**Cardiovascular diseases** ^*§*^	Present	127 (22.4)	3 (4.5)	0.003	6.07 (1.88–19.67)	
	Absent	439 (77.6)	63 (95.5)		1	
**Dermatological symptoms** ^*¥*^	Present	112 (19.8)	4 (6.1)	0.006	3.82 (1.36–10.73)	3.66 (1.07–12.55)
	Absent	454 (80.2)	62 (93.9)		1	1
**Adverse obstetric outcomes**^*¶*^ **(n = 572)** ^*+*^	Present	191 (36.8)	9 (17.0)	0.004	2.85 (1.36–5.96)	2.45 (1.08–5.57)
	Absent	328 (63.2)	44 (83.0)		1	1

* Logistic regression analysis adjusted for age, literacy level, occupation, socioeconomic status and passive smoking

^#^ diminished vision, cataract, eye irritation and watering of eyes

^$^ Throat irritation, ear pain, asthma, nasal stuffiness/ running nose, cough with/ without phlegm

^§^ Hypertension, myocardial infarction, and stroke;

^¥^skin irritation, blisters due to skin burns;

^¶^ miscarriages, still birth, low birth weight and preterm deliveries;

^+^among those who were ever married

The association of cumulative exposure to biomass fuel in hour-years and health status of women was studied. The variable biomass in hour-years was computed by multiplying the number of hours of exposure to biomass fuel per day and the number of years of exposure to the same. The maximum total exposure to biomass in hour-years among women was estimated and further categorized into tertiles based on the study data as following—none (*0 hour-years)*, mild (0.1 - ≤ *30 hour-years)*, moderate (*30*.*1–78*.*0 hour-years)* and severe exposure (≥*78*.*1 hour-years*). As the cumulative exposure to biomass in hour-years increased, there was a statistically significant increase in self-reported ophthalmic conditions (p<0.001), respiratory symptoms (p<0.001), cardiovascular diseases (p<0.001), dermatological symptoms (p <0.001) and history of adverse obstetrical outcomes (p<0.05).

## Discussion

In the present study, almost half of the participants were ≤ 45 years of age (49.5%) compared to a study in Tamil Nadu where 66% of the participants were less than 50 years old [21]. A study in Odisha reported a higher proportion of the rural women to be illiterate (36.2%) and homemakers (94.8%) compared to our study population (6.6% and 79.1% respectively). However, majority of the study participants were Hindu by religion (80.8%), which corroborates with our study findings (91.8%) [16].

In our study, 53.1% of the surveyed families had a separate room for cooking within their houses and 10.6% had an outdoor kitchen while Agrawal S et al. who analysed NFHS-III data inferred contrast findings (45.9% and 16.7% respectively) [32]. Johnson P. et al. also reported a higher proportion of families (50.7%) to be using outdoor kitchen compared to our study [21].

In this study, prevalence of biomass fuel use for cooking was 72.5%, which is higher compared to the prevalences reported by the Census of India 2011 (India—66.3%, Karnataka State—61.5% and Udupi district-64.9%) [26, 33, 34]. However, according to the sub-district data of rural Udupi, 70.4% of the households utilize biomass, which is similar to our study findings [7]. A report from rural districts of Andhra Pradesh, India found that the prevalence of using traditional stoves without a smoke outlet in the families ranged from 18.6–74.7% while in our study 63.8% of the biomass users did not have a smoke outlet [28].

In our study, 61.0% of the women were exposed to biomass fuel for more than 22 years and 76.7% used the fuel for almost two hours (more than 90 minutes) per day. Likewise, majority of the women in rural Tamil Nadu, India cooked using biomass fuel for ≥ 15 years (73.2%) and almost two-third of them used it for more than two hours per day (63.3%) [21]. Biomass fuel is cheaper and easily available. These were the two common reasons quoted by our study population for continuing its use, which is similar to the findings reported in a study in Ethiopia and Kenya [35, 36].

Associations between socio-demographic factors and exposure to biomass fuel was studied and it was found that women who were aged above 45 years, those with low literacy levels (0–5 years of schooling), engaged in unskilled occupations or homemakers and those from low socio-economic status were significantly associated with biomass fuel use in their lifetime. An increasing trend of using firewood was observed with increasing age in Malawi, which ranged from 33% among women of the age 17–24 years, approximately 45% among 46–55 years to 56.3% between 66–85 years (p< 0.001) [8]. A study in Sri Lanka reported that having less than 10 years of schooling (p<0.05) as well as lower family income (p<0.05) were associated with biomass fuel use which is in line with our observations (p< 0.001) [9].

In the present study, we observed that women with ophthalmic conditions were more likely to be exposed to biomass fuel (OR = 3.90; 95% CI: 2.31–6.61). Pokheral A.K. et al. observed a significant association between current biomass usage and the development of nuclear cataracts (OR = 2.58; 95% CI: 1.22–5.46), which also increased with the duration of exposure in years [37]. Similar findings were also reported by Ravilla T.D. et al. (AOR = 1.28; 95% CI: 1.10–1.48) [38]. In contrast, Kazi A. found no such associations between ophthalmic conditions and biomass use among women [39].

It was observed that women with respiratory symptoms had five times higher risk of being exposed to biomass fuel (OR = 5.04; 95% CI: 2.52–10.07) compared to those without symptoms, which is similar to the findings reported in Mexico and in India [40, 18]. A systematic review by Sana A. et al. also concluded that COPD was more likely to be diagnosed among women who had a history of exposure to biomass fuel (OR = 1.38; 95%CI: 1.28–1.57) [41]. However, a study in Pakistan found no such associations [39].

The present study showed that women with self-reported cardiovascular symptoms/conditions were at six times higher risk of being ever exposed to biomass fuel and similar findings were reported by a study in Odisha, India (p<0.05), in Nigeria (OR = 1.67; 95% CI: 1.56–4.99) and in China (OR = 2.58; 95% CI: 1.53–4.32) [16, 42, 43].

In our study, we observed that women with a history of adverse obstetric outcomes were almost three times (OR = 2.85; 95% CI: 1.36–5.96) more likely to be biomass fuel users as compared to those without a bad obstetric outcome which supports the findings reported by Samaraweera et al., (OR = 3.83; 95% CI: 1.50–9.90), Tipre M. et al., Haider M.R. et al. and Alexander D.A. et al. [3, 44, 45, 46].

To summarize, more than two-thirds of the families were using biomass in our study and it was found that being ever exposed to biomass fuel was significantly associated with their socio-demographic characteristics, self-reported ophthalmic, respiratory, dermatological, cardiovascular symptoms /conditions and history of adverse obstetric outcomes. However, on multivariable regression analysis, exposure to biomass was positively associated with self-reported ophthalmic, dermatological symptoms and history of adverse obstetric outcomes. Our study findings also suggest that households using both LPG and biomass fuel for cooking may have serious health implications from exposure to mixed fuel use. Therefore, these comparatively wealthy households may require continued public health interventions such as subsidies and regular health education.

Given the cross-sectional nature of this study, role of the biomass fuel exposure in adverse health outcomes of the women needs to be confirmed further by longitudinal studies in this region. Other limitations of the study include logistic constraints due to which the clinical diagnosis of the symptoms reported by the women could not be made during field visits and the duration of health conditions, information regarding use of fuels in years as well as the time spent near the biomass fuel could be subjected to recall bias.

## Conclusion

The study findings provide baseline information regarding prevalence of biomass fuel use for cooking at household level, the association of biomass fuel use with socio-demographic characteristics and self-reported health conditions among the women given the paucity of such existing data in southern part of Karnataka, India. Based on the study findings, it is recommended that community awareness programs should be organized to promote the use of appropriate smoke outlets for biomass stoves wherever it is used. Further longitudinal studies ascertaining the levels of household harmful air pollutants using an air quality monitor as well as research studies focusing on the effectiveness of interventions such as effect of improved cook stove use on health status of the women are required in this region. Reducing IAP will enable us to achieve SDG-3 by reducing maternal mortality, mortality due to communicable and non-communicable diseases and this can be attained by providing affordable, accessible and cleaner fuel (in alignment with SDG-7 and SDG-13) to the families, which is the need of the hour [47].

## Supporting information

S1 Data Study questionnaire. (PDF)

S2 Data Study dataset. (SAV)
