# Supplementary material for: Household cooking fuel use and its health effects among rural women in southern India—A cross-sectional study
Source: PLoS One. 2020 Apr 27;15(4):e0231757. doi: 10.1371/journal.pone.0231757 (PMC7185712; doi:10.1371/journal.pone.0231757)
Supplement: S1 Data — (PDF) [file pone.0231757.s001.pdf]

MAIN QUESTIONAIRE

A.GENERAL INFORMATION –

A1. Family ID: ________________

A2. Locality: ____

A3. Socioeconomic status: High/Medium/Low

A4.Religion: ______________

A5.Total income of the family:_______________

B.COOKING PRACTICES

B1. Where do you cook usually?
a) In a room used for living or sleeping (with partition)
b) In a room used for living or sleeping (without partition)
c) In a separate room used as kitchen
d) In a separate building used as kitchen
e) Outdoors

B2.Descibe the use of each stove in the house

**Purpose-Currently used**	**Most importantly used stove ***	**Second most commonly used stove ***	**Average hours of use (most important stove)**	**Average hours of use (Second most important stove)**
Cooking for home				
Cooking food/drink for selling outside				
Heating water				
Cooking animal feed				

* a) Biomass portable open stoves without chimney
b) Biomass fixed open stove without chimney
c) Biomass portable stove with chimney   d) Biomass fixed stove with chimney
e) Kerosene Stove   f) LPG stove   g) others

B3. How long does it take to cook one meal?
a) Breakfast ______
b) Lunch _______
c) Dinner _______
d) Others (Specify) __________

C FUEL USE

C1.What type of fuel do you use for the following purposes?

Purpose	Fuel used *		Duration of usage in years		Average use in hours		Reason for using the fuel**	
	Fuel1	Fuel2	Fuel1	Fuel2	Fuel1	Fuel2	Fuel1	Fuel2
Cooking for home								
Cooking food/drink for selling outside								
Heating water								
Cooking animal feed								

*a) Wood (logs) b) Crop Residues c) Dung Cakes d) Coal
e) Charcoal f) Kerosene g) LPG h) Bio-Gas
i) Electricity j) Solar cooker k) Others__________

**a) More affordable b) Easily available c) The fuel is of better quality
d) The fuel produces less smoke e) Takes lesser time to cook
f) Others _______________

C2a. Do you use kerosene lamps? Yes/No
C2b.For how long have you been using kerosene lamps? _____________

C3. After use of biomass, what is the method of disposal preferred?

a) Dumping
b) Controlled tipping or sanitary land fill
c) Incineration
d) Composting
e) Burial
f) Municipality bins

C4.Does the household ever changed its fuel use pattern? Yes/ No

C5.If yes the fuel changed from ________ to _________.

C6.The reason for change :

D.HABITS

D1a. Does anyone smoke in your household? Yes / No

D1b.List the people who smoke in the family, the number of cigarettes/bidis and for how may years

Sl.no	Household member	Number of cigarettes/day	Duration (Years)	Number of bidis/day	Duration
1					
2					
3					
4					
5					
6					

D2.Where do you and/or other family members (if any) primarily smoke?

a) Inside the house
b) Outside the house
c) Both inside and outside the house
d) Other________________

D3a.Do you burn incense sticks in your house? Yes /No
D3b.How many incense sticks do you burn per day? ___________________

D4a.Do you burn mosquito coils in your house? Yes/No
D4b. How many mosquito coils do you burn per day? __________________

E.HOUSING CHARACTERISTICS

E1. Type of house
a) Kucha
b) Pucca
c) Mixed

E2.Is there overcrowding present in the household? Yes/No

E3.How is the ventilation of the house?
a) Adequate
b) Inadequate

E4.How is the lighting of the house?
a) Adequate
b) Inadequate

F.KITCHEN / COOKING AREA CHARACTERISTICS

F1. Kitchen/cooking area dimensions:
a) Length: _______ feet
b) Width: _______ feet
c) Height: _______ feet

F2.How is the ventilation of the kitchen?
a) Adequate
b) Inadequate

F3.How is the lighting of the kitchen?
a) Adequate
b) Inadequate

F4.Type of permanent ventilation present in the cooking area/kitchen?
a) Gaps in between roof and the wall
b) Windows
c) No ventilation

F5.Are the windows open while cooking? Yes/No

F6.Is the doors open while cooking? Yes/No

F7.Is there a smoke outlet /exhaust fan present in the kitchen? Yes/No

F8.Is the open air kitchen enclosed on all sides? Yes/No

G.HEALTH STATUS

G1.Does anyone in the family suffer from tuberculosis? Yes/No

G2.Have they received medical treatment for tuberculosis? Yes/No

G3.If yes, what was the outcome?
a) Declared cured
b) Failure
c) Incomplete treatment
d) Relapse

QUESTIONNAIRE

A.GENERAL INFORMATION

A1.Individual ID ____________

A2.Age:_____________

A3.Literacy status:________________________

A4.Occupation:______________________

A5. Cooking status:

a) Involved in cooking at present
b) Not involved in cooking at present but at past have been involved in cooking
c) Exposed to cooking fuel use but never involved in cooking

A6. At what age did you start cooking? ____________

A7.For how long have you been cooking? __________

A8.Which type of fuel you have been using since the time you started cooking?

**Purpose**	**Fuel used ***		**Duration of usage in years**		**Average use in hours**		**Reason for using the fuel****	
	**Fuel1**	**Fuel2**	**Fuel1**	**Fuel2**	**Fuel1**	**Fuel2**	**Fuel1**	**Fuel2**
Cooking for home								
Cooking food/drink for selling outside								
Heating water								
Cooking animal feed								

*a) Wood (logs) b) Crop Residues c) Dung Cakes d) Coal
e) Charcoal f) Kerosene g) LPG h) Bio-Gas
i) Electricity j) Solar cooker k) Others__________

**a) More affordable b) Easily available c) The fuel is of better quality
d) The fuel produces less smoke e) Takes lesser time to cook
f) Others _______________

A9a.Do you use kerosene lamps? Yes/No
A9b.For how long have you been using kerosene lamps? _____________

B.HEALTH STATUS

Sl. no	Disease/ Symptom	Have you experienced the disease /symptoms ?	Frequency of episodes per year	Duration	Received treatment	Was hospitalization required any time?
1	Diminished vision	Yes/No			Yes/No	Yes/No
2	Cataract	Yes/No			Yes/No	Yes/No
3	Throat irritation	Yes/No			Yes/No	Yes/No
4	Eye irritation	Yes/No			Yes/No	Yes/No
5	Watering of eyes	Yes/No			Yes/No	Yes/No
6	Skin Burns/Blisters	Yes/No			Yes/No	Yes/No
7	Skin irritation	Yes/No			Yes/No	Yes/No
8	Ear pain	Yes/No			Yes/No	Yes/No
9	Cardiovascular diseases	Yes/No			Yes/No	Yes/No
10	Hypertension	Yes/No			Yes/No	Yes/No
11	Asthma	Yes/No			Yes/No	Yes/No
12	Chronic bronchitis	Yes/No			Yes/No	Yes/No
13	Cough without phlegm	Yes/No			Yes/No	Yes/No
14	Cough with phlegm	Yes/No			Yes/No	Yes/No
15	Nasal Stuffiness/ Running nose	Yes/No			Yes/No	Yes/No
16	Others _______________	Yes/No			Yes/No	Yes/No

B19a.Do/ Did you suffer from tuberculosis? Yes/No
B19b. If yes, have you received medical treatment for tuberculosis? Yes/No
B19c.If yes, what was the outcome?

a) Declared cured
b) Failure
c) Incomplete treatment
d) Relapse

B20.How many children do you have? ___________

B21a.Do you have a history of miscarriages? Yes/No
B21b.How many miscarriages did you have? ___________
B21c. At which month did you have the miscarriage? ___________

B22a.Do you have a history of still births? Yes/No
B22b.How many stillbirths did you have? ____________
B22c.At what month did you have stillbirth? ____________

B23a.Did your children have a history of IUGR at time of pregnancy? Yes/No
B23b.How many children had a history of IUGR? ____________

B24a.Did your children have a history of low birth weight at time of delivery? Yes/No
B24b.How many children had a history of low birth weight? ____________
B24c.What was the birth weight of the child? ____________

B25a.Did you have a preterm delivery? Yes/No
B25b.How many children were born preterm? ____________
B25c.At what month was your child born preterm? ____________
